# Chordoma in the lateral medullary cistern in a patient with tuberous sclerosis: A case report and review of the literature

**DOI:** 10.4103/2152-7806.63908

**Published:** 2010-05-31

**Authors:** Kristopher T. Kimmell, Hayan Dayoub, Ethan D. Stolzenberg, Eric H. Sincoff

**Affiliations:** College of Medicine, The University of Oklahoma Health Sciences Center, Oklahoma City, OK, USA; 1 Department of Neurosurgery, The University of Oklahoma Health Sciences Center, Oklahoma City, OK, USA; 2 Department of Pathology, The University of Oklahoma Health Sciences Center, Oklahoma City, OK, USA

**Keywords:** Chordoma, Cytogenetics, Medullary cistern, Tuberous sclerosis

## Abstract

**Background:**

Chordomas are rare intracranial tumors. There are several reported cases of these tumors arising in patients with tuberous sclerosis (TSC), a neurocutaneous disorder inherited in autosomal dominant fashion that predisposes patients to hamartomatous and neoplastic lesions.

**Case Description:**

A 38-year-old man with the diagnosis of TSC presented with the complaint of dizziness and near syncope. Imaging revealed a mass in the lateral medullary cistern that was found at the time of surgery to be a chordoma. The patient underwent a left far lateral approach for removal of the tumor. Upon opening of the dura, the tumor could be seen under the arachnoid. The tumor was carefully debulked within the limits of safety. The patient did well postoperatively and was referred to the radiation oncology department at our institution for follow-up radiotherapy of the tumor bed.

**Conclusion:**

This study presents an unusual presentation and location for a chordoma and contributes to the growing literature associating chordomas with TSC.

## INTRODUCTION

Chordomas are identified as rare tumors. They represent <1% of all intracranial neoplastic processes.[30] They are derived from remnants of the notochord and typically develop along the spinal column primarily in the sphenooccipital region cranially and the sacrococcygeal region caudally. Tuberous sclerosis (TSC) is a neurocutaneous disorder inherited in autosomal dominant fashion. This disorder predisposes patients to develop a number of hamartomatous as well as neoplastic processes. There is a growing body of literature associating chordomas with TSC.[2,9,12–14,22,26,28] In this study, we present the case of a 38-year-old man with the diagnosis of TSC that developed a chordoma in the lateral medullary cistern. This study represents further evidence for a possible association between TSC and chordomas.

## CASE DESCRIPTION

**Figure 1:**
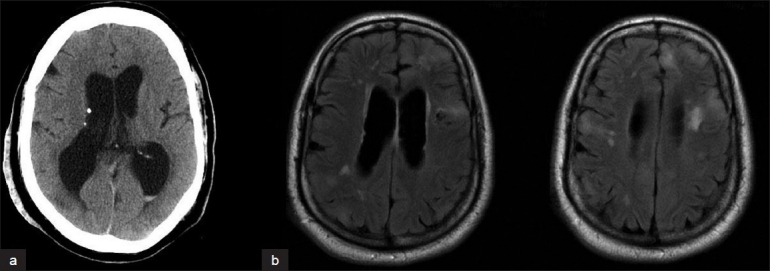
(a) CT brain showing periventricular calcifications and (b) MRI FLAIR sequence showing subcortical and periventricular white matter changes. Both of these findings are common in patients with a diagnosis of tuberous sclerosis

**Figure 2:**
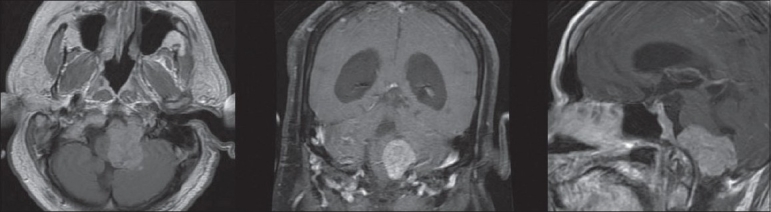
Preoperative MRI of the brain T1-weighted postcontrast infusion sequences in axial, coronal, and sagittal planes demonstrating an extra-axial, eccentrically shaped mass with contrast enhancement in the left aspect of the medullary cistern compressing and displacing the brainstem and cerebellum to the right

A 38-year-old man presented to the emergency room at our institution with the complaints of dizziness and a near-syncopal episode. This episode was preceded by a 1-week history of nausea, vomiting, and dysarthria. He also reported a 2-month history of new headache. The patient denied any visual disturbances, hearing changes, recent seizures, loss of consciousness, weakness, or sensory changes. Medical history was significant for a diagnosis of TSC with mild mental retardation and seizures in the distant past as well as hypertension. The patient had had a cataract removed remotely. Physical examination revealed adenoma sebaceum on the patient's malar surfaces. Complete neurological examination revealed no focal deficits. The results of laboratory tests were within normal ranges. A computed tomography (CT) of the head from an outside hospital demonstrated a brain mass with high attenuation. The mass appeared adjacent to the left cerebellar hemisphere near the Foramen of Lushka and extending into the foramen magnum. The patient had mild hydrocephalus evidenced by the prominence of his ventricular system. Periventricular calcifications were consistent with his diagnosis of TSC [Figure 1a]. The patient was admitted for work-up of this brainstem mass. Magnetic resonance imaging (MRI) of the brain with contrast demonstrated deep cortical white matter changes typical of patients with TSC [Figure 1b]. MRI also showed an extra-axial, irregularly shaped, well-marginated mass with heterogeneous enhancement in the left aspect of the medullary cistern with displacement of the brainstem and cerebellar peduncle to the right [Figure 2]. Further careful review of the imaging showed that the majority of the clivus also demonstrated uptake and enhancement of contrast. Increased T2 signal in the brainstem and cerebellum was consistent with edema. The patient was administered steroids and discharged with a plan to return to the hospital for elective removal of the brain mass.

**Figure 3:**
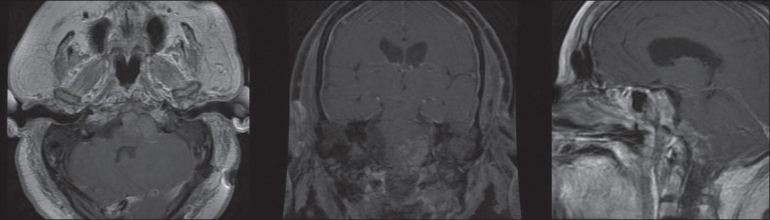
Postoperative MRI of the brain T1 postcontrast infusion sequences in axial, coronal, and sagittal planes demonstrating extent of surgical debulking

The patient was taken to the operating room for a left far lateral approach for removal of the tumor. Upon opening of the dura and release of cerebrospinal fluid, the tumor could be seen under the arachnoid and appeared to originate intradurally. Several cranial nerves were adherent to the mass, eliminating the possibility of a total resection. During the operation, no connection between the intradural mass and the clivus could be appreciated. The tumor was carefully debulked within the limits of safety. A significant amount of cerebellar swelling was encountered during the case and ultimately led to early termination of the operation before subtotal resection could be completed. The patient was then transferred to the ICU. He remained intubated but was following commands with all four extremities. Postoperative MRI showed significant reduction of tumor burden from surgical debulking [Figure 3]. 

**Figure 4:**
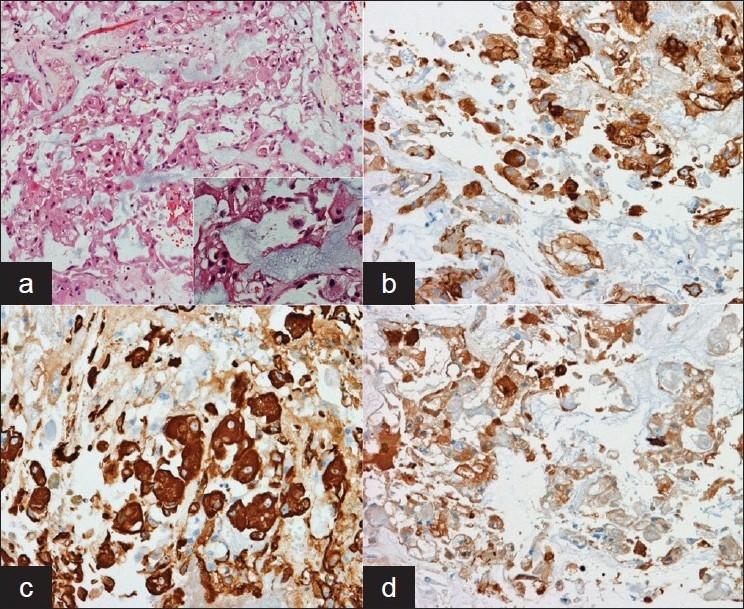
(a) On medium-magnification, eosinophilic neoplastic cells can be seen in the midst of a mucoid matrix; Inset: high-powered magnification shows markedly foamy cytoplasm in some neoplastic cells (the so-called physaliphorous cell). Immunohistochemical staining for (b) epithelial membrane antigen, (c) Pan-cytokeratin, and (d) S-100 demonstrate positive staining for all three in the neoplastic cells. This pattern of immunohistochemical staining, coupled with the histologic characteristics of the tumor, are consistent with a chordoma of the skull base.

Grossly, the tumor was gray-tan in appearance. Microscopic examination of the tumor revealed plump, vacuolated cells within a mucinous matrix [Figure 4]. Tumor cells were positive for pan-cytokeratin (pan-CK) and epithelial membrane antigen (EMA), focally positive for S-100, and negative for glial fibrillary acidic protein (GFAP) and neurofilament (NF). Less than 1% of nuclei labeled positively for Ki-67. These histologic and immunohistochemical staining characteristics were consistent with a diagnosis of chordoma. This diagnosis was unexpected given the location of the tumor both within the lateral medullary cistern as well as under the arachnoid.

Postoperatively, the patient developed dysphagia and required placement of a percutaneous endoscopic gastrostomy (PEG) tube as well as a tracheostomy. The patient also developed a pneumonia, which was treated with moxifloxacin on the recommendation of the infectious disease team. The patient's pneumonia resolved and his dysphagia improved, and he was discharged with plans to follow up with radiation oncology for radiotherapy of his residual tumor.

## DISCUSSION

Chordomas are found to be rare tumors that are believed to arise from remnants of the notochord. About 45-50% of chordomas are reported to arise in the sacrococcygeal region; 35-40% in the sphenooccipital region; the remaining 15% of these tumors arise along the rest of the spinal column.[11,12,14,22,25,28,30] The typical age range of patients presenting with these tumors is 30-50 years old for intracranial tumors and 40-60 years old for sacrococcygeal lesions.[15] Intracranial chordomas classically arise in the midline in the region of the clivus. Patients can present with headache, nausea, vomiting, and cranial nerve palsies.[30] These tumors are normally very locally aggressive, often showing extensive surrounding bony erosion. Distant metastasis is an unusual occurrence, although there is a report in the literature of a chordoma of the clivus presenting with cutaneous manifestations from metastasis.[14] 

Radiographically, chordomas are well-defined extra-axial masses that enhance with contrast on CT.[22,30] The tumors may demonstrate focal calcifications. On MRI, the tumors are typically iso- to hypo-intense on T1-weighted imaging and hyperintense on T2-weighted imaging and typically show heterogeneous enhancement with the addition of contrast.[22] This heterogenous pattern of enhancement helps to distinguish chordomas from other common tumors of the skull base such as meningiomas and schwannomas that normally have a more homogenous pattern of enhancement.[15] 

Pathologically, chordomas appear grossly gray to tan in color with gelatinous texture and are often lobulated and encapsulated.[22,30] They can be divided into chondroid and nonchondroid subtypes on the basis of the evidence of chondroid differentiation within the tumor.[30] Histologically, they show a pattern of cells with foamy cytoplasm in a mucinous matrix (the characteristic physaliphorous cells). Immunohistochemical staining will often demonstrate positive staining for cytokeratin and epithelial membrane antigen (EMA) with variable staining for vimentin (in cases of chordomas with chondroid differentiation) and S-100. The epithelial phenotype, expressed in positive staining for cytokeratin and EMA, is important in differentiating chordomas with chondroid features from chondrosarcoma.[12] 

Tuberous sclerosis is a neurocutaneous disorder inherited in an autosomal dominant fashion. It's prevalence is estimated to be 1:6800.[20] Clinically, the disease is characterized by a number of manifestations, including depigmented macules (ash leaf spots), raised plaque-like flesh colored lesions (shagreen patches), and adenoma sebaceum, which are fibroangiomatous nevi typically found on the malar prominences of the face. Classically, the central nervous system is affected in the disease by cortical tubers (from which the disease derives its name), which are hamartomatous, calcified nodules found in the deep cortical and periventricular white matter. Patients may also demonstrate focal areas of demyelination at the grey-white junction.[26] These patients often develop seizure disorders and can have varying degrees of mental retardation.

Tuberous sclerosis is believed to involve two separate genes; TSC1, which is found on the long arm of chromosome nine (9q32–q34) and which encodes the protein product hamartin, and TSC2 found on the long arm of chromosome 16 (16q13.3) that encodes for the protein tuberin. Both of these proteins are believed to play a role in tumor and growth suppression. Because of the dysfunction of these gene products, TSC patients are at risk of developing a number of tumors. The classic central nervous system tumor associated with TSC is the subependymal giant cell astrocytoma (SEGA), which arises in 10% of patients with TSC. These tumors usually arise within the ventricles.[26] Although the location of the tumor in this patient would have been atypical of a SEGA, based on his diagnosis of TSC and the intradural location of the tumor, the initial clinical suspicion was a SEGA and the frozen section was read as such. However, as already outlined, the permanent section and immunohistochemical profile of the tumor were more consistent with the diagnosis of chordoma.

Although SEGA is the tumor classically associated with TSC, there are a number of case reports of chordomas arising in patients with this genetic disorder[2,9,12–14,22,26,28] [Table 1]. In several cases, the TSC patients have unusual presentations for a patient with a chordoma both in terms of location as well as age. The seminal case report of a chordoma in a TSC patient was by Dutton *et al*., who presented a newborn male with sacrococcygeal chordoma, thick sclerotic ribs, and thoracic and abdominal aortic aneurysms.[9] There are several reports of chordomas in TSC pediatric patients. Kombogiorgas *et al*. reported on a chordoma involving the clivus in a 15-week-old male baby with family history of TSC and imaging features consistent with this diagnosis.[12] The previously mentioned metastatic chordoma with cutaneous lesions was a case of a clivus chordoma arising in a 20-month-old baby with TSC.[14] Storm **et al**. reported on a chordoma arising in the cervical region in a 16-year-old patient with a history of TSC.[28] Schroeder **et al**. presented another clivus chordoma in a 4-year-old female baby with TSC,[26] and Borgel **et al**. reported on a clivus chordoma in 4-year-old boy with family history of TSC.[2] Lee-Jones **et al** reported on sacral chordomas in two patients, one a 33-week-old female fetus electively aborted due to cardiac abnormalities diagnosed by prenatal ultrasound and the other a newborn female.[13]

**Table 1:** Chordomas reported in patients with tuberous sclerosis

Report	Age	Gender	Location
Dutton **et al**.[9]	Term newborn	M	Sacrococcygeus
Schroeder **et al**.[26]	4 years	F	Clivus
Poskitt **et al**. [22]	4 years	F	Clivus
Borgel **et al**.[2]	4 years	M	Clivus
Lee-Jones **et al**.[13]	33-week fetus	F	Sacrococcygeus
Kombogiorgas*et al*[12]	15weeks	M	Clivus
	Term newborn	F	Sacrococcygeus
Lountzis **et al**.[14]	20 months	M	Clivus—with cutaneous and other metastases
Storm **et al**.[28]	16 years	F	Cervical spine
Current report	38 years	M	Lateral medullary cistern

**Table 2:** Cytogenetic studies in chordomas

Study	Number of tumors	Tumor location	Cytogenetic abnormalities identified
Bayrakli **et al**.[1]	7 primary; 11	Clivus	Gains—1q25 (66.6%), 1p36 (60%), 7q33 (37.5%), 4q26–q27 (12.5%), 3p12–p14 (10%) for
	recurrences	(all primary)	recurrent tumors; 7q33 (33.3%), 3p12–p14 (16.6%), 1q25 (14.2%), 1p36 (14.2%) for primary
			tumors
			Losses—1q25 (66.6%), 2p13 (55.5%), 1p36 (30%), 17p13.1 (20%), 6p12.5%), 3p12–p14
			(10%) for recurrent tumors; 2p13 (83.3%), 6p12 (50%), 1q25 (32.7%), 1p36 (28.5%),
			3p12-p14 (16.6%), 7q33 (16.6%), 17p13.1 for primary tumors
Bridge **et al**. [3]	1 primary	Sacrum	42,XY, add(1)(p11),−3,der(4)t(4;?;?18;?)(q12;?;?;?),−6,−9,der(9)t(6;9)(q11;p11), −(14),der(16)
			t(4;?;16)(q12;?;q11),der(17)add(17)(p12)t(17;18)(q11;p11),der(18)del(18)(p11)add(18)(q?)
Buonamici **et al**.[5]	3 primary; 1 recurrence	3 clivus; 1 cervical	All 3 clivus—normal karyotype; cervical recurrence−46,XY[15]/46,XY,t(6;11)(q12;q23)[5]
Butler **et al**.[6]	5 primary	Lumbosacral	Normal karyotypes in 4/5; random abnormalities in one cell of fifth
Dalpra **et al**.[7]	2 recurrences (same patient)	Clivus	39,XY,dic(1;9)(p36.1;p21),add(1)(p12),del(3)(p13),−4,−4,der(6)t(1;6;14)(6pte→ 6q27::1p36→ 1 p13::14pter→ 14qter),−7,−8,−8,−11,−17,−17,−18,−18,−19,−20,−20,−22,−22,+8mar
DeBoer **et al**.[8]	1 primary	Sacrum	43,XY,−2,−3,del(4)(q32),−6,+7,−11,der(12)t(9;12)(q12;p11),add(16)(q23),−20,add(22)
			(q13),+mar.
Gibas **et al**..[10]	2 primary	Sacral	36,X,−X,−1,−3,−4,−10,−11,−13,−14,−18,der(21)t(1;21)(q21;q22),−22114]
			72,XX,−X,+1,del(1)(p22)x2,−2,−3,add(3)(p25),−4,del(5)(p13),add(5)(p15),add(5)(p13),−7,inv(7)
			(qllq22},add(9)(p24)x2,−10,−10,−10,+12,−13,−13,add(15)(p11),−17,add(18)(p11),add(19)
			(q13),+20,add(20)(q13)x2,der(21)t(2;21){q11;q22)x2,+9mar[25]
Lee-Jones *et al*.[13]	2 primary	Sacral	1/2—TSC2 (16q13.3)
			1/2—TSC1 (9q32−q34)
Mertens *et al*.[17]	6 primary, 3 recurrences	Sacrum	1/9−42,XY,add(1)(p31),del{2)(p21),−3,add(3)(p11),−4,t(5;7)(q33;q36),add(8)(24),del(9)
			(p13),−10,add(11)(q11),dup(12)(q13q24),−16,−18,ins(18;?)(q21;?),add(19)(p13),der(22)t(4;22)
			(q11;p11), +mar[3]/46,Y,
			del(X)(q24),t(1;5)(p36;q33),del(2),der(3)t(3;14)(p21;q24)t(X;3)(q24;q11),der(6)
			t(3;6)(p21;p21),del(9),−10,add(11),del(12)(q13q15),+add(12)(q24),der(14}
			t(3;14}(q21;q24},der(15}t(6;15)(p21;p13}, −16,?add(17)(p11),add(19), +
			mar[4]/48,XY,del(2),der(2)t(2;?;12)(p14;?;q13),inv(4)(p16q31),add(5)(p15),+7,+8,del(9),−
			10,del(11)(p12),del(12),add(16)(p13),add(17)(q21),add(19},+mar[4]
			1/9−46,XY,t(1;6)(q44;q11) [5]/46,XY[20]
			1/9−40,XY,der(1)t(1;21)(pll;q11),−3,−4,−8,der(8)t{1;8)(q21;q23),add(9)(q22), −13,−14,der(20)
			t(2;20)(q21;q13),del(2)(q35),−21113]/77−84,idemx2,+3,+8,+2mar[6]/46,XY[3]
			All others-normal karyotype
Miozzo. *Int J Cancer.*	Two	Clivus	See Dalpra *et al*
2000	recurrences		
Persons *et al. Cancer*	Two	Sacrum	1/2—normal karyotype
*Genet Cytogenet.*	recurrences		1/2—44,XY,t(1;3)(q42;q11),−2,der(7)t(2;7)(q23;q32),−21 and 46,X,t(Y;8)(q12;q22),t(1;14)
1991			(p34;q32),t(5;10)(q13;p11)
Sawyer *et al*.	11 primary,	19 clivus, 3	*Case 1*
*Neurosurg Focus.*	11	cervical	46,XX,inv(1)(q23q42),t(1;10)(q32;p11),t(3;14)(p21;q13),inv(4)(p14q31),add(12)(q22),del(14)
2001	recurrences		(q32)[5]
			46−48,XX,add(1)(q?32),del(3)(p25),del(5)(q31),del(6)(q15),add(11)(p13),+del(12)(q22),-
			13,add(16)(p11),add(16)(q24),+17,der(18)t(1;18)(q12;q23)x2,add(19)(q13)[cp3]
			46,X,del(X)(p22.1),t(1;9)(p36.1;p13),t(4;9)(p12;q34),t(6;16)(p11;q24)[2]
			45,X,del(X)(p11.2p11.4),der(5)t(5;14)(p13;q11),?add(11)(q22),del(12)(q22),−13,der(17)
			t(13;17)(q14;p13),add(21)(q22)[3]
			*Case 2*
			48,XY,+5,+7,+12,−13,add(13)(q34),−18,+20[2]
			49,idem,+19[16]
			*Case 3*
			44−45,XY,t(2;14)(p23;q11),?t(3;12)(p21;p13),del(4)(q?23),−5,−6,der(11)t(6;11)(q11;p12)[cp2]
			*Case 4*
			46,XX,t(4;17)(q23;q21),t(8;9)(q11;q11)[3]
			46,XX,t(2;20)(p31;p11.2),t(3;22;16)(p21;q11.2;q22),del(6)(p23)[2]
			*Case 5*
			52−66,−X,−X,−Y,add(1)(q22)x2,i(1)(q10),+2,−3,der(3)t(3;4)(p?24;q?13)t(1;3)(q21;q21)
			X2,−4,der(4)del(4)(p12)add(4)(q13)x2,add(6)(q?21)+7,−9,−10, add(11)(q13)x2,add(11)
			(q25),+add(11)(q25),−12,−
			13,add(13)(?q13),?del(15)(q11.1q13)x2,?dup(15)(q11.1q13),add(17)(q24),−18,+19,−
			20,+21,+22[cp9]
			46,XY,1,der(1)?t(1;4)(p13;q?27),+der(3)t(3;11)(q29;q11)add(3)(p13),del(4)(p12),der(4)
			add(4)(p16)t(1;4), inv(7)(p?21q?34),add(8)(p22),del(9)(q21),del(10)(q22),der(11)add(11)
			(p15)?del(11)(q13q22),?inv(14)(q11.2q32),+add(15)(q22),der(15;16)(q10;q10),?t(17;20)
			(q?23;q?13.3)[cp4]
			*Case 6*
			38,X,−X,i(1)(q10),−3,−4,add(6)(q27),+7,−9,−10,−13,−14,−18,−22[cp5]
			*Case 7*
			43−45,−Y,del(X)(q?26),t(2;17)(q21;p13),del(6)(q?25),add(9)(p24),inv(14)(q13q24)[cp10]
			46−47,XY,t(1;2)(q44;q13),inv(3)(p?24.2q29),t(4;9)(q33;q22),t(8;16)(q24.1;q24),t(10;13)
			(p13;q12)[cp5]
			*Case 8*
			44−46,XX,t(1;22)(p32;q11.2),?t(2;20)(q33;q11.2),der(3)t(3;4)(p21;q?31;1)t(3;22)
			(q21;q13),der(4)t(3;4),?t(9;15)(p22;q22),t(17;18)(q21;q23)[cp15]
			*Case 9*
			45−47,Y,del(X)(p22.1),del(1)(q24),?t(8;18)(q24.1;q23),add(9)(q34),?inv(9)(p11q22),del(16)
			(q?22),add(17)(p11),add(19)(q13),del(20)(q11.2)[cp20]
			*Case 10*
			45−46,XY,der(1)t(1;1)(q42;p34.1),der(1)t(1;1)t(1;14)(q24;q11.2),t(3;5)(p23;p11),t(3;10)
			(q21;q24),t(4;6)(q31.1;p11.2),t(4;16)(q25;p11.2),der(?6)del(6)(p12p21.2)t(6;12)
			(q?27;?21.2),t(13;16)(q12;q22),der(14)t(1;14)[cp10]
			*Case 11*
			46,X,?del(X)(q26q26),inv(3)(p23q?25),inv(7)(p22q22),add(8)(p23),der(8)t(8;9)(p11.2;q22)
			t(8;13)(q13;q11),der(9)t(8;9),t(10;15)(q26;q12),der(13)t(8;13),del(17)(p11.2p11.2),t(21;22)
			(q11.2;q13)[cp11]
			46,XX,inv(20(p13q11.2),del(7)(q11.1q11.2),t(14;19)(q11.2;p13.3),del(16)(q23)[2]
			46,der(X)del(X)(p11.4)del(X)(q21),der(X)t(X;X)(q26;p11.4),der(5)t(5;9)(q11.2;q32),inv(6)
			(p23q?13),der(7)t(7;9)(p15;p24),t(7;15)(p12;q15),t(8;12)(q24.1;q22),?t(8;19)(p22;p11),der(9)
			t(7;9)(p15;p24)t(5;9)(q11.2;q?32),t(10;20)(p13;q11.2),del(22)(q13)[3]
			37−40,XX,i(1)(q10),−3,?t(3;13)(p26;q11),−4,?t(4;5)(p14;q33),der(5)t(?4;5)(q?21;q?33),−
			6,+7,der(9)add(9)(p24)del(9)(q22),−10,−13,−14,del(15)(q21),−18,add(20)(p11.2),−
			22,+mar[cp3]
Scheil *et al*. Genes	7 primary, 9	10 sacrum,	*Case 1*—Gains: 7; 8p;9q34; 12q34; 15q; 17, 20 q. Losses: 1p34−p21; 3; 10; 11; 14q; 18; 22
Chromosomes	recurrences	5 clivus, 1	*Case 2*—Gains: 7q36, 20. Losses: 1p31.3−p22; 3p21−p12; 13q21−q32; 18q22−q23
Cancer. 2001		spinal	*Case 3*—Gains: 1p34.2−p36; 7p21−qter; 12p; 15q; 22q. Losses: 1p31−p21; 3p; 6q11−q21; 9p;
			Xp
			*Case 3(2)*—Gains: amp1p34.2−p36; 7; 12p; 15q; 22q. Losses: 1p31−p21; 2q33−q36; 3p;
			6q11−q21; 9p−q31; Xp
			*Case 4*—Gains; 7q22−qter; 12p
			*Case 4(2)*—Gains: 7q22−qter. Losses: 3; 4; 5; 9p; 10
			*Case 5*—Gains: 17; 20q. Losses: 1p31; 4p; 9p21−p24; 13q21
			*Case 6*—Gains: 5q23−qter; 7; 12q24; 20. Losses: 3; 4q35
			*Case 6(2)*—Gains: 5q31−qter; 7q34−qter; 12q24; 20; 22q; Xq23−qter
			*Case 7*—Gains: 1q, 3p, 4q12−q27, 5q, 7, 8pter−q21.1, 8q24, 1q, 3p, 4q12−q27, 5q, 7,
			8pter−q21.1, 8q24
			*Case 8*—Gains: 20; 21q22; 22q; Xp, Xq26−q28
			*Case 9*—Gains: 11q24−q25; 12q24; 14q21−qter; 17q; 20q; 21q21−q22. Losses: 6q; 12p; 13q
			*Case 10*—Gains: 7q34−qter; 20q. Losses: 1p31−p21; X
			*Case 11*—Gains: 12q24. Losses: 13q21−q31; Xq25−Xqter
			*Case 12*—Gains: 1q; 11q24−q25. Losses: 1p; 3; 4; 9p; 10; 13q; 14q; X
			*Case 13*—Gains: 5p15; 7q34−qter; 9q34; 22q. Losses: 3p12−p14; 13q; 18q

It is worth noting that in many of these cases, TSC patients presented with chordomas at a very early age, much younger than is typical for this tumor in the general population. Furthermore, while several of the patients had chordomas arise in locations typical of this tumor, others have had tumors present in more eccentric locations, such as with cutaneous metastasis, or, in the case of our patient, in a more posterolateral position within the skull base. Certainly, chordomas can arise in atypical locations. Lu *et al* reported on a patient with a chordoma arising in Meckel's cave.[15] Kaufman *et al*. presented a chordoma in a 9-year-old male child arising in the region of the jugular foramen.[11] 

An unusual characteristic of the case reported by Kaufman *et al*., and shared by our case, is that the tumor was found and appeared to originate intradurally. Although the MRI of our patient demonstrated the enhancement of the clivus, the primary tumor that was symptomatic was found lateral to the medulla. Typically, chordomas arising from the clivus are midline and extradural in location. However, Kaufman *et al*. point out that there are cases of intradural chordomas, and proffer an explanation as to why, embryologically, this could be possible. They suggest that these chordomas represent neoplastic transformation of an ecchordosis physaliphora, rests of notochord cells found intradurally and reported in about 2% of all autopsies.[4,16] They also suggest that the lateral location of a chordoma may be a result of rostral forking of the notochord, a phenomenon that has been previously reported.[23] Nevertheless, a chordoma arising intradurally is an unusual recurrence, and there are several reports of intradural chordomas arising in the midline[16,19,31] as well as the lateral skull base.[11,29]

There is evidence to suggest a genetic influence in the development of chordomas. Stepanek *et al*. analyzed pathological specimens of four chordomas arising in one pedigree that suggested an autosomal dominant pattern of inheritance.[27] Dalpra *et al*. performed cytogenetic analysis on a chordoma in a patient who had a family history of chordoma.[7] There have been several recent reports examining the cytogenetics of chordomas[1,3,5–8,10,13,17,18,21,24,25] [Table 2]. Bayrakli *et al*. in their study of 7 primary tumors and 11 recurrences, highlighted four genetic loci (1p36, 1q25, 2p13, and 7q33) affected in primary chordomas as well as recurrences.[1] Dalpra *et al*.'s study also identified cytogenetic changes in chromosome 1p in their analysis of two recurrences of a clival chordoma.[7] They later mapped this abnormality to 1p36, one of the same loci highlighted in the Bayrakli study.[18] Several other reports lend support to the tumor suppressor role of chromosome 1p in chordomas.[3,17,24,25] Gibas *et al*. identified a cytogenetic abnormality in chromosome 21q22 in their analysis of two primary sacral chordomas. Nearly every chromosome has been highlighted as having additions, deletions, hypo- or hyper-diploidy in the above referenced studies.

While all of these studies have highlighted various cytogenetic abnormalities in the tumors they have analyzed, to date no consistent cytogenetic abnormality has been identified in any of the chordomas. Furthermore, there is no indication that any of the chordomas analyzed in these studies reported from patients with TSC. The only study to specifically look at the cytogenetics of chordomas arising from patients with TSC was Lee-Jones *et al*.[13] They subjected two cases of sacral chordomas in TSC patients to cytogenetic analysis. They found germ-line mutations of TSC1 in one case and TSC2 in the other case, with somatic inactivation of the wild-type allele within the tumors.[13] These findings, coupled with the growing number of cases of chordomas in patients with TSC, suggest a possible relationship, and a better understanding of the tumor biology in this patient population would be beneficial.

## CONCLUSION

Chordomas are rare intracranial neoplasms. They are typically located midline, extradurally, in the sphenooccipital region. Rarely, these tumors may be found intradurally or laterally in the skull base. This study represents an unusual presentation of a chordoma arising in the lateral medullary cistern. Furthermore, this case joins several case reports suggesting a link between chordomas and TSC, a neurocutaneous disorder predisposing patients to neoplastic processes. Further studies need to be done to elucidate the relationship between tuberous sclerosis and chordomas.
